# Sun-basking fish benefit from body temperatures that are higher than ambient water

**DOI:** 10.1098/rspb.2018.0639

**Published:** 2018-05-30

**Authors:** Oscar Nordahl, Petter Tibblin, Per Koch-Schmidt, Hanna Berggren, Per Larsson, Anders Forsman

**Affiliations:** Department of Biology and Environmental Science, Linnaeus University, Barlastgatan 11, 392 31, Kalmar, Sweden

**Keywords:** aquatic, basking behaviour, ectotherm, fish, thermoregulation

## Abstract

In terrestrial environments, cold-blooded animals can attain higher body temperatures by sun basking, and thereby potentially benefit from broader niches, improved performance and higher fitness. The higher heat capacity and thermal conductivity of water compared with air have been universally assumed to render heat gain from sun basking impossible for aquatic ectotherms, such that their opportunities to behaviourally regulate body temperature are largely limited to choosing warmer or colder habitats. Here we challenge this paradigm. Using physical models we first show that submerged objects exposed to natural sunlight attain temperatures in excess of ambient water. We next demonstrate that free-ranging carp (*Cyprinus carpio*) can increase their body temperature during aquatic sun basking close to the surface. The temperature excess gained by basking was larger in dark than in pale individuals, increased with behavioural boldness, and was associated with faster growth. Overall, our results establish aquatic sun basking as a novel ecologically significant mechanism for thermoregulation in fish. The discovery of this previously overlooked process has practical implications for aquaculture, offers alternative explanations for behavioural and phenotypic adaptations, will spur future research in fish ecology, and calls for modifications of models concerning climate change impacts on biodiversity in marine and freshwater environments.

## 1. Introduction

Temperature plays a key role in defining spatial distributions, temporal activity patterns and bodily functions of species, populations and individuals [1–4]. Fish can optimize development, growth and reproduction by fine-tuning body temperature through behavioural thermoregulation, most notably habitat selection [1,2]. Some species of fish, such as tunas (Scombroidei), sharks (Lamniformes) [5]; and the opah (*Lampris guttatus*) [6], can evade the temperature boundaries of ambient water by generating and conserving metabolic heat internally. This capacity coincides with niche expansion and increased swimming performance, but is restricted to only 0.1% of all known species of fish [5]. In terrestrial environments, cold-blooded animals can attain higher body temperatures by sun basking, and thereby potentially benefit from broader niches, improved performance and higher fitness [7–10]. The higher heat capacity and thermal conductivity of water compared with air have been universally assumed to render heat gain from sun basking impossible for aquatic ectotherms, such that their opportunities to behaviourally regulate body temperature are largely limited to choosing warmer or colder habitats [11–14]. There are reports of fish that expose themselves to sunlight in the surface layer [13,15,16]. However, to our knowledge, the consequences of such aquatic sun basking have not been systematically evaluated, and it has not been previously established whether sun basking enables fish to become warmer than the surrounding water.

Here we explore whether the thermal rewards of sun basking previously demonstrated to be of utmost importance in terrestrial environments are also available to organisms living in water. To that end, we first performed experiments with temperature loggers and cylindrical fish-like physical models in different settings. Next, to quantify the thermal consequences of sun basking in free-ranging live fish, we fitted carp (*Cyprinus carpio*) with data storage tags (DSTs) and monitored basking behaviour, body temperature and water temperature during two summer months. We also investigated whether differences in temperature excess among individuals were associated with coloration and behavioural boldness. Finally, to assess the potential for any thermal benefits associated with basking to influence individual performance, we investigated whether the magnitude of the body temperature excess gained during basking was associated with growth rate.

## 2. Material and methods

### (a) Operative temperatures and physical models

To test for an effect of sunlight and colour on the temperature of objects submerged in water, we performed two field experiments using temperature loggers (HOBO Pendant Temperature/Light 64 K, Onset Computer Corporation, 58 × 33 × 23 mm, 18 g). We used a design with 10 pairs of temperature loggers in each experiment, carried out outdoors under natural light conditions in a small experimental pond (see below). Each pair was attached to a floating raft that kept the loggers at a fixed depth (4 ± 2 mm) and distance from each other (18 cm). Two squares of Styrofoam (19 cm × 8 cm × 2 cm) joined by two crossbars (diameter = 3 mm) formed the raft and both temperature loggers were positioned horizontal on two parallel bars (diameter = 1 mm) attached to the underside of the two squares of foam. In the first experiment, exposure to sunlight was manipulated by sheltering one of the loggers in each pair with a cover (12.5 cm wide, 23 cm long, and 5 mm thick) of isolating foam lined with reflective aluminium foil on the upper side to remove any effect of sunlight on temperature gain of shaded loggers. The sun-exposed loggers were not covered. The loggers were painted with black acrylic paint (CRC Ind, ProPaint, black matt). The rafts were placed in the pond, individually anchored and incubated during 2 days (9–10 July 2017) with calm and sunny conditions prior to data extraction.

In a second experiment performed to test for an effect of colour on heat gain, the temperature loggers used in the first experiment were stripped from the black paint and then repainted either black or white (CRC Ind, ProPaint, white matt) to ensure the same thickness of paint. A paired design was used with one black and one white temperature logger without covers placed on each of the ten rafts spread out into the pond, individually anchored and incubated during 2 days (13–14 July 2017) with calm and sunny conditions prior to data extraction. We next compared the magnitude of the effect of colour on heat gained by sun basking in aquatic as opposed to terrestrial environments. To that end, five rafts with submerged black and white temperature loggers were placed and incubated in the pond, and another five rafts with loggers were placed on land and incubated simultaneously (15 July 2017) under identical solar and weather conditions.

All loggers were calibrated after the experiments by incubation in water bath at 20°C, 23°C and 26°C (1 h per temperature regime). Mean differences between loggers within pairs during calibration were small (after experiment one: 0.01 ± 0.07°C; after experiment two: 0.00 ± 0.07°C), but were nevertheless used to correct the data from the experiments prior to analyses.

To estimate warming up rates and the range of excess body temperatures potentially available to sun basking fish, we quantified operative temperatures with physical models [17] under different conditions. Physical models (*n* = 4) of fish were made from large (length = 20 cm, diameter = 5 cm) and small (length = 12 cm, diameter = 2 cm) plastic cylinders filled with silicone and covered with either black or white adhesive super duct tape (Stokvis Tapes, China, 31-3417-1, and 31-3417-2). Each model was equipped with a micro-thermocouple placed in the centre of the cylinder and connected to a quick reading digital thermometer (Fluke model 52 K/J, John Fluke Mfg. Co. Everett, WA). The models were mounted horizontally on a raft side by side, separated by roughly 5 cm, and exposed to artificial light (Toplite 250 W halogen; HIE 250 dw E40 coated, positioned roughly 50 cm above the models). In separate trials, models were submerged just below (approx. 0.5–1 cm) the water surface in plastic tanks (1000 l filled to approx. two-thirds) at high (21°C) water temperature, at low (13°C) water temperature, or placed above water/in air (approx. 22°C room temperature), on 27–30 January 2015. Two additional micro-thermocouples placed next to the physical models but without being exposed to direct light were used to record temperature of surrounding water or air, respectively. To validate the process under natural light conditions the experiment with physical models was repeated outdoors in a sheltered, shallow (depth approx. 2 m) bay (lat. 56.710071, long. 16.365885) of the Baltic Sea, on a day (12 June 2015) with sunny and calm conditions, using a similar set-up as in the laboratory trials.

### (b) Field study on carp

After assessing the potential of the mechanism using operative temperatures of the physical models, a field study was performed to evaluate the role and importance of sun basking for live and free-ranging fish in the wild. For this experiment, we used 48 *Cyprinus carpio* carps (supplied by Aneboda Fiskodling, Sweden; mean length = 28.7 cm, range 24–32 cm) that represented either a natural dark (*n* = 25) or a pale orange (*n* = 23) colour morph. To quantify the difference between morphs we compared dorsal lightness from digital images of fish that had been sedated (water bath with benzocaine [50 mg l^−1^]) and photographed (Canon EOS 1000D, Shutter 1/13, F4.5, ISO 400) under artificial light (Osram L 18 W/840 Lumilux, Cool White). The photos were analysed according to the standardized colour space CIELAB using Adobe Photoshop. A mean value of lightness, L*, for the dorsal region was extracted for each fish.

Fish were tagged with surgically implanted data storage tags (DSTs, Lat 1410, Lotek Wireless Inc.) that logged core body temperature, ambient water temperature and depth every 5th minute. The tags had two temperature sensors: one internal placed in the body cavity and one external at the tip of flexible stalk outside of the fish going through the abdominal wall anterolateral of the anal fin and ending below the tail (caudal) fin (see [6] for a similar approach with the same type of tag), thus enabling estimation of excess body temperature for each individual fish.

After recovering from tagging and behavioural assessment, carps were released (8 June 2016) in a man-made clay pond (lat. 57.173382, long. 16.032958, size approximately 70 × 20 × 1.7 m) for a duration of two months. The only other fish species in the pond was crucian carp (*Carassius carassius*). A high feeding activity by a dense population of crayfish (*Pacifastacus lenisculus*) was observed in the middle of the study period and sinking carp pellets were provided as supplementary food on a daily basis thereafter (from 15 July 2016) to reduce any effects on carp basking behaviour of competition from crayfish. The pond was surrounded by deciduous trees, resulting in partial and moving shade on the pond surface. Three avian species known to prey on carp, the grey heron (*Ardea cinerea*), the great cormorant (*Phalacrocorax carbo*) and the osprey (*Pandion haliaetus*), were observed foraging in the pond during the field period. Upon termination of the field study, carp were recaptured from the pond by repeated seine-haul fishing during three days (9–12 August 2016).

Fish were assessed for boldness (see below) and measured for body length (measuring board, to the nearest cm) both before and after the field period. Sex was determined by visual inspection of gonads when the tags were removed.

### (c) Boldness

A standard emergence test was used to score the fish according to a shyness–boldness continuum [18]. This was done to determine if individuals were consistent in boldness over the study period and to test for associations of behaviour with colour morph, sex and body temperature. The test arena consisted of a tank (h 30 cm × w 79 cm × l140 cm) with aerated freshwater (≈19°C) and illuminated by artificial daylight (250 W, 18000 lumens, HIE 250 DW Coated). A black shelter box (h 33 cm × w 27 cm × 63 cm) was placed in the short end of the rectangular tank with a front opening facing the other end of the tank. The opening of the shelter box was sealed with a removable guillotine type door. Each assessment trial started so that the fish was released into the shelter box where it was left to acclimatize for 10 min before the guillotine door was raised giving the fish access to the arena. A video camera (GoPro HERO4) located above the tank was used to record the behaviour of the fish. Trials were terminated 35 min after opening the door of the shelter box. Time until the fish emerged from the shelter box (with the entire body outside the box) and started to explore the arena was extracted from the video recordings and used as a measure of boldness. Boldness was assessed on three occasions following the same procedure: (i) prior to surgical tagging with DSTs; (ii) two weeks after tagging and prior to release into the pond; and (iii) upon termination of the study after having spent eight weeks in the pond.

### (d) Statistical approach

**Figure 1.**
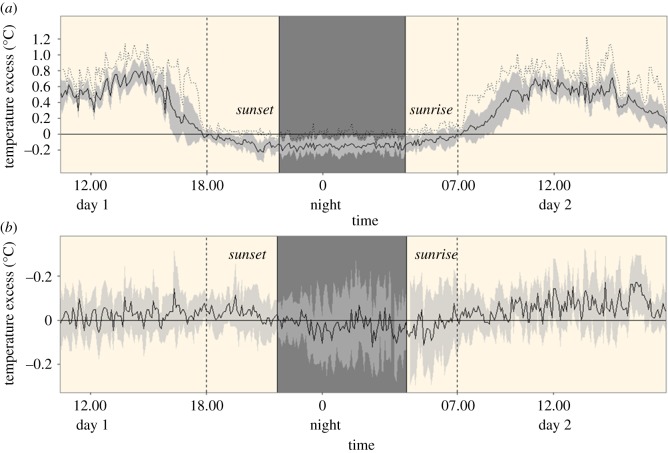
Daily temperature excess of data loggers submerged in water according to sun exposure and colour. (*a*) Mean (solid line) ± standard deviation (shaded area) temperature excess of sun-exposed compared with shaded loggers (*n*_pairs_ = 10) along with maximum values (dotted line) during two days in the pond. (*b*) Mean ± standard deviation (shaded area) temperature excess of black compared to white loggers (*n*_pairs_ = 10). Dark and pale background denotes night and day respectively. Black loggers were significantly warmer than white loggers during intense sun exposure (07.00–18.00 h) on day two (paired *t*-test, *t*_9_ = 3.10, *p* = 0.013) but not on day one (*t*_9_ = 0.72, *p* > 0.05). The two experiments were performed during different dates. (Online version in colour.)

In the operative temperature experiments, effects of sun exposure and colour on temperature of submerged data loggers were evaluated using separate paired *t*-tests (difference within pairs compared to zero). These analyses were based on calculations of day and night mean values for sun exposed and shaded loggers, and from black and white loggers, respectively. Results from the first experiment showed that sun exposed loggers were warmer than shaded loggers during the period 07.00 h to 18.00 h (figure 1*a*). In the analysis the effect of colour was restricted to this time interval.

In the field study, 42 of the 48 DST tagged carp originally released in the pond were recaptured but 14 had lost their tags and two were found dead. For the analysis of basking behaviour (depth) and body temperature, DST data were used from 10 June to 8 August, excluding days close to release and recapture of the carp. To analyse fish temperature data we performed general linear mixed models implemented using the procedure MIXED in SAS [19,20]. Temperature excess during basking was treated as the dependent variable, colour morph (natural dark or pale orange) as a fixed factor with two levels, time period of day (morning 06.00–10.59; midday 11.00–15.59; and evening 16.00–20.59) as a fixed factor with three levels, and their interaction as a fixed factor with three levels. Individual identity was included as random factor to evaluate and account for dependence and greater similarity of repeated temperature observations within than among individuals. Date was also included as a random factor, to account for any variance in temperature excess among days that might occur as a result of varying weather conditions. The Kenward–Roger method was used to approximate degrees of freedom [20]. Statistical significance of fixed effects was assessed using the *F* statistic [19]. Statistical significance of the random factor individual was assessed using the Wald *Z* test [19,20]. However, quantification and formal testing for random effects of date was not necessary for evaluating the main hypotheses under investigation and the associated parameter estimates are therefore not reported. Data for males and females were analysed separately because of the huge number of observations in the dataset and to avoid problems associated with over-parametrizing of the statistical models due to inclusion of higher-order interactions.

For most analyses, we only included data from the DSTs recorded during sunny conditions because we were interested in evaluating the effects of sun basking on body temperature during exposure to light. Periods of sunny conditions (defined as light intensity greater than 600 W m^−2^) were identified using data of sunlight intensity at the pond obtained from the STRÅNG database from the Swedish Meteorological and Hydrological Institute. Basking periods were further defined by depth and time: uninterrupted sequences of surface position (upper approximately 35 cm of the water column) that lasted longer than 20 min. Data from the physical models (see above) demonstrate that it takes approximately 20 min for temperature excess to build up. Because an effect of sun basking is not likely to manifest until after this initial lag phase, temperature data from basking periods that were shorter than 20 min were excluded. For the same reason, temperature data for the first 20 min were excluded from basking periods. Temperature data during night (21.00–05.59) were excluded. A baseline drift in temperature excess was detected for the DSTs, and this was corrected prior to statistical analysis by using temperature excess during night time as a baseline.

To further evaluate whether body temperature rises due to sunlight, we included in one analysis also data from the DSTs recorded during cloudy conditions and tested for a difference in temperature excess between cloudy and sunny conditions. Data for males and females were analysed separately using the procedure MIXED in SAS, as described above, and least-squares means were used for the comparison between cloudy and sunny conditions.

Data on time to emergence from the shelter box obtained in the three behavioural assessment trials were used to evaluate individual consistency in boldness over the duration of the study. Using the package rptR [21] for R, a mixed model was fitted with sex, colour morph and trial number as fixed effects while the individual was treated as a random effect. Adjusted repeatability, which controls for fixed effects, was extracted for the random effect individual. Confidence interval for the repeatability estimate, *r*, was acquired by bootstrapping 1000 times.

Data on mean emergence time from the first two boldness trials that were performed before the fish were released in the pond were used to analyse whether boldness differed between colour morphs. The Wilcoxon two-sample test was used, and data for males and females were analysed separately. The mean emergence time values were also used to test whether temperature excess was associated with boldness. To this end, an analysis of covariance was performed using procedure GLM in SAS. In this analysis, temperature excess during basking was treated as the dependent variable, sex and colour morph were treated as fixed factors, and boldness (time to emergence) was treated as a covariate.

We evaluated whether the increase in body temperature gained from sun basking translated into differential performance of individuals. To that end, we tested for an association of growth (length gain during the eight weeks spent in the pond) with temperature excess using correlation analysis.

## 3. Results and discussion

### (a) Data loggers and physical models

To assess whether it is possible for aquatic animals such as fish to attain temperatures in excess of ambient when exposed to sunlight and test if colour affects the rate and magnitude of temperature gain by basking we first performed experiments with temperature loggers and physical models (see Material and methods for details). Data loggers that were submerged in water close to the surface and exposed to sunlight became warmer compared with shaded loggers during the day (*t*_9_ = 18.80, *p* < 0.001; figure 1*a*). Furthermore, black-painted loggers attained higher temperatures than white loggers during sun exposure (figure 1*b*). The effect of colour (0.17°C; peak value of mean excess; figure 1*b*) corresponded to about 22% of the difference between sun exposed and shaded black loggers (peak value of mean excess; figure 1*a*).

To further quantify the potential for temperature gain of basking fish, we estimated operative temperatures [17] using cylindrical fish like physical models. Results indicated that fish may need to remain dormant in the surface for about 20 min to benefit from development of temperatures in excess of the ambient water and further corroborated that dark coloration enhances the rate and magnitude of heat. The effects of radiation and colour on heat gain and excess temperatures of submerged objects were independent of water temperature, and qualitatively similar but of lower magnitude (approx. 1.5°C *viz*. 5–20°C depending on size) than in air (figure 1; electronic supplementary material, figures S1–S3).

### (b) Sun basking in free-ranging carp

**Figure 2.**
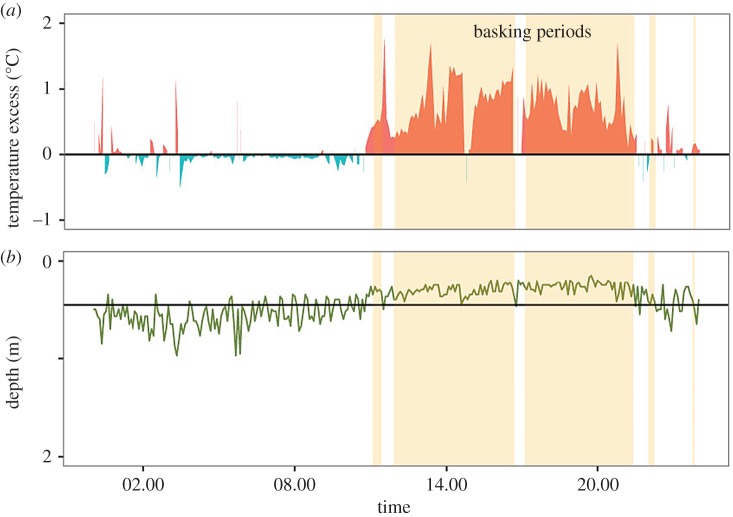
Daily temperature excess and depth profile for one sun basking carp. Data for 1 day, 25 June 2016, showing (*a*) a marked increase in temperature excess during sun-basking periods, which were defined as (*b*) dormancy in the surface layer (above the horizontal line; basking event depicted in orange in both panels).

**Figure 3.**
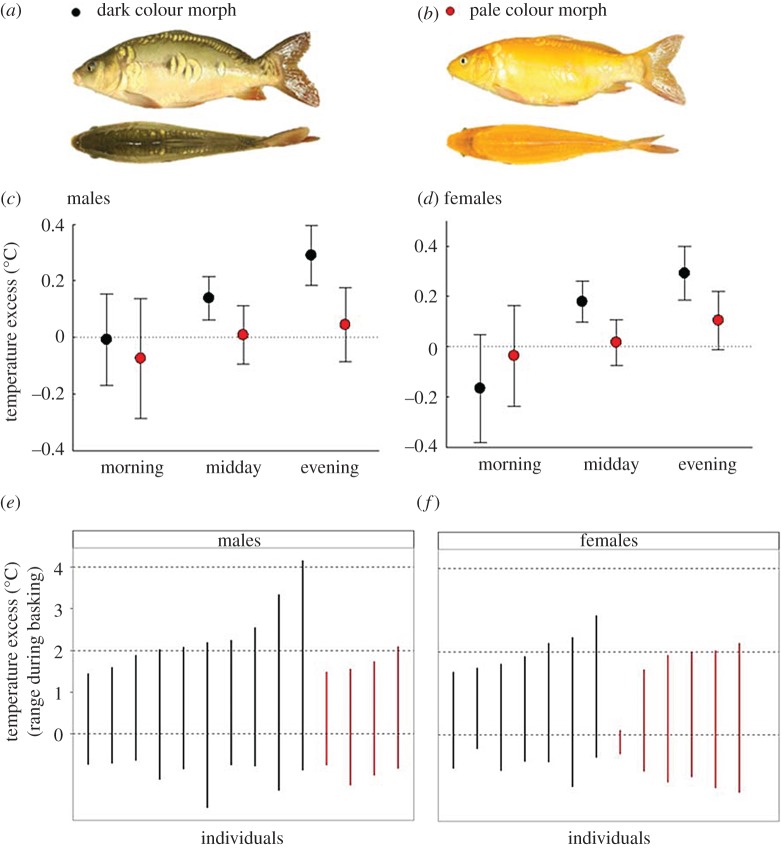
Variation in body temperature excess of sun-basking male and female carp depends on colour morph and period of day. (*a*,*b*) Dark and pale colour morphs of carp. (*c*,*d*) Least-squares means ± 95% confidence intervals obtained from general mixed model ANOVAs (see electronic supplementary material, table S1) in which colour morph (black or orange), period of day (morning 06.00–10.59; midday 11.00–15.59; and evening 16.00–20.59) and their interaction were treated as fixed effects and individual identity, and date was treated as random effect. Data for males (*n* = 14, left panel) and females (*n* = 13, right panel) were analysed separately. Dotted horizontal lines indicate that body temperature is equal to the temperature of the surrounding water. Results from analyses of daytime observations of surfacing fish during sunny conditions. (*e*,*f*) Ranges of temperature excess for individual male (left panel) and female (right panel) carp. Black and red bars represent data for dark and orange individuals.

While physical models are valuable for assessing the potential of natural processes, care must be taken to extrapolate from models to real organisms [22]. We therefore next assessed the effects of sun basking, coloration and behaviour on body temperature in free ranging live fish under natural conditions. To that end, we used carp (*Cyprinus carpio*), a benthic omnivorous cyprinid species that is important in aquaculture [23], targeted by recreational fishing [24], widely depicted in art and used as ornaments in garden ponds owing to the highly variable colour pattern [25]. We fitted carp with data storage tags (DSTs) that monitored basking behaviour (periods of dormancy in the surface layer) and its effect on temperature excess (body temperature minus water temperature; figure 2) during two summer months (8 June to 8 August 2016) in an experimental pond. Data from the tags in recovered individuals that belonged to a dark brown or a pale orange colour morph (figure 3*a–b*) showed that carp attained core body temperature in excess of ambient water temperature during basking (figure 2), and that this effect depended on time of day and colour morph (electronic supplementary material, table S1). Mean temperature excess was at the highest at midday and evening, while there was no difference between body and ambient water temperature during morning (figure 3*c–d*). The temperature excess ranged up to 4°C higher than ambient water temperature (figure 3*e–f*). A comparison based on data collected during days with different weather conditions showed that temperature excess was significantly higher on sunny days than on cloudy days, in both males (least-squares means (95% CI) obtained from mixed model ANOVAs: cloudy: 0.030 (−0.012 to 0.073), sunny: 0.056 (0.012 to 0.099), *p* < 0.0001) and females (cloudy: 0.032 (−0.010 to 0.075), sunny: 0.068 (0.024 to 0.111), *p* < 0.0001). These findings provide rare evidence that fish exposed to sunlight in the surface layers can benefit from body temperatures exceeding ambient water temperature. In our study the DSTs were located in the abdominal cavity while it is the dorsal side that is heated by radiation. Our results may therefore underestimate the benefits of sun basking, particularly with regard to the dorsal musculature and the head region.

### (c) Association of body temperature with colour pattern

A dark coloration increases thermoregulatory efficiency and allows for higher body temperatures in terrestrial ectotherms [9,12,26,27]. The results from data loggers and physical models, together with comparisons between the two colour morphs of carp (figure 3), show that the same principle applies in water. Fish categorized as belonging to the brown morph were darker (lightness, L*[mean ± s.d.]: 132 ± 21) than fish representing the orange morph (187 ± 4; ΔL*[lightness] >50; two sample *t*-test, *t*_8_ = 5.66, *p* < 0.001). The difference in darkness seemed sufficient to impact the efficiency at which solar radiation is absorbed and converted to body heat because dark individuals attained higher temperature excess during basking than orange individuals during midday and evening (electronic supplementary material, table S1; figure 3*c–d*). That separate analysis of males and females yielded similar results strengthens reliability of this observation. The present findings conform well to those from studies of ectotherms in terrestrial environments [9,26,28], but to our knowledge an association of body temperature with colour pattern has not previously been demonstrated in fish or other aquatic animals.

### (d) Association of body temperature with personality

Sun-basking behaviour reflects a compromise between competing needs of temperature regulation, seeking refuge from predators and foraging [8,9,26,28]. Dormancy in the surface layer exposes sun-basking fish not only to radiant energy, but also to potential predators that soar above or lurk beneath the surface. Being benthic feeders, carp probably surface with temperature regulation as a primary objective. Tendency to take risks is often correlated over a range of contexts, known as behavioural syndromes [29,30]. We therefore examined whether body temperature varied among individuals according to differences in personality. Repeated assessments of individuals along a shyness–boldness continuum [18] showed that differences in time to emergence from a shelter box were consistent over the time frame of the pond study (*R* = 0.269, 95% CI [0.03; 0.54], *p* < 0.05), and bolder individuals reaped higher temperature rewards of basking compared with shy individuals (electronic supplementary material, table S2 and figure S4). However, orange individuals did not gain more heat than dark brown individuals despite being bolder (electronic supplementary material, figure S4), which can probably be attributed to the more efficient conversion of radiant energy to body heat in darker individuals.

### (e) Body temperature excess and growth rate

**Figure 4.**
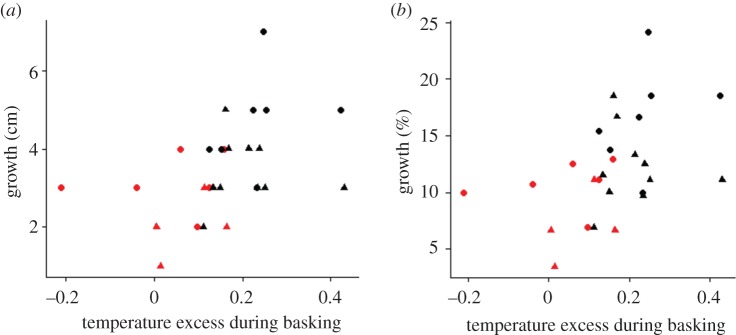
Relationship between temperature excess during sun basking and growth. Growth (body length gain during eight weeks in a pond) increased with temperature excess (absolute growth (*a*): *r* = 0.42, *n* = 27, *p* = 0.0284; *r*_s_ = 0.52, *n* = 27, *p* = 0.0056; relative growth (*b*): *r* = 0.44, *n* = 27, *p* = 0.0217, *r*_s_ = 0.51, *n* = 27, *p* = 0.0072). Figure shows mean values for individuals. Colour and shape of symbols indicate pale orange (red) and dark (black) colour morphs of female (circles) and male (triangles) carp.

Any body temperature gained by basking will be of ecological and evolutionary significance only insofar as it impacts on performance and fitness of individuals. The increase in body temperature during basking was modest (1–4°C increase), but even differences in the order of 1°C translate into 6–10% change in rate of most physiological processes [4,7,31]. To obtain direct evidence for effects of sun basking on performance in carp we analysed changes in body length of individuals during the pond study. Overall, growth increased with increasing temperature excess during basking (figure 4). Given the profound importance of growth rate and body size for survival, fecundity and longevity [32,33], this indicates that aquatic basking can confer fitness advantages in fish.

## 4. Conclusion

We propose basking in aquatic environments as a previously unrecognized mechanism for fish to obtain temperatures in excess of ambient water. Our results show that objects submerged in water can attain temperatures in excess of ambient water temperature when exposed to sunlight, and that the normally bottom-dwelling carp can use this effect by basking near the surface and thereby benefit from faster growth. The thermal benefit was higher for individuals with a dark dorsal coloration, which offers a new explanation to countershading in fish, and many other animals; a phenomenon that has traditionally been interpreted as an adaptation that improves camouflage [34]. Besides dark colour, aquatic basking does not necessarily require physiological adaptations, large body size or a high metabolic activity to elevate body temperature, and it can thus be a widespread behaviour with potential to influence spatio-temporal distributions of fish. Thermal benefits aside, basking at the surface may allow for parasite removal via symbiotic skin-cleaning relationships [16]. Sunlight is also known to have beneficial effects on microbial infections and various skin diseases [35]. We envision that aquatic basking will have practical implications for productivity in aquaculture, spur future research in fish ecology ranging from physiology, via behavioural ecology to evolutionary biology, and improve models concerning how global warming will impact biodiversity and ecosystem functioning in marine and freshwater environments.

## Supplementary Material

Electronic Supplementary Results

## Supplementary Material

Data S1

## Supplementary Material

Data S2

## Supplementary Material

Data S3

## Supplementary Material

Data S4

## Supplementary Material

Data S5

## Supplementary Material

Data S6

## Data accessibility

The datasets supporting this article are accessible via the electronic supplementary material (data S1–S6).
