# Supplementary material for: Sun-basking fish benefit from body temperatures that are higher than ambient water
Source: Proc Biol Sci. 2018 May 30;285(1879):20180639. doi: 10.1098/rspb.2018.0639 (PMC5998101; doi:10.1098/rspb.2018.0639)
Supplement: Electronic Supplementary Results [file rspb20180639supp1.pdf]

Electronic Supplementary Material

Sun basking fish benefit from body temperatures that are higher than ambient water

Oscar Nordahl, Petter Tibblin, Per Koch-Schmidt, Hanna Berggren, Per Larsson and Anders Forsman

Proceedings R Soc B

This file includes: Supplementary Figures S1 to S4 Supplementary Tables S1 to S2

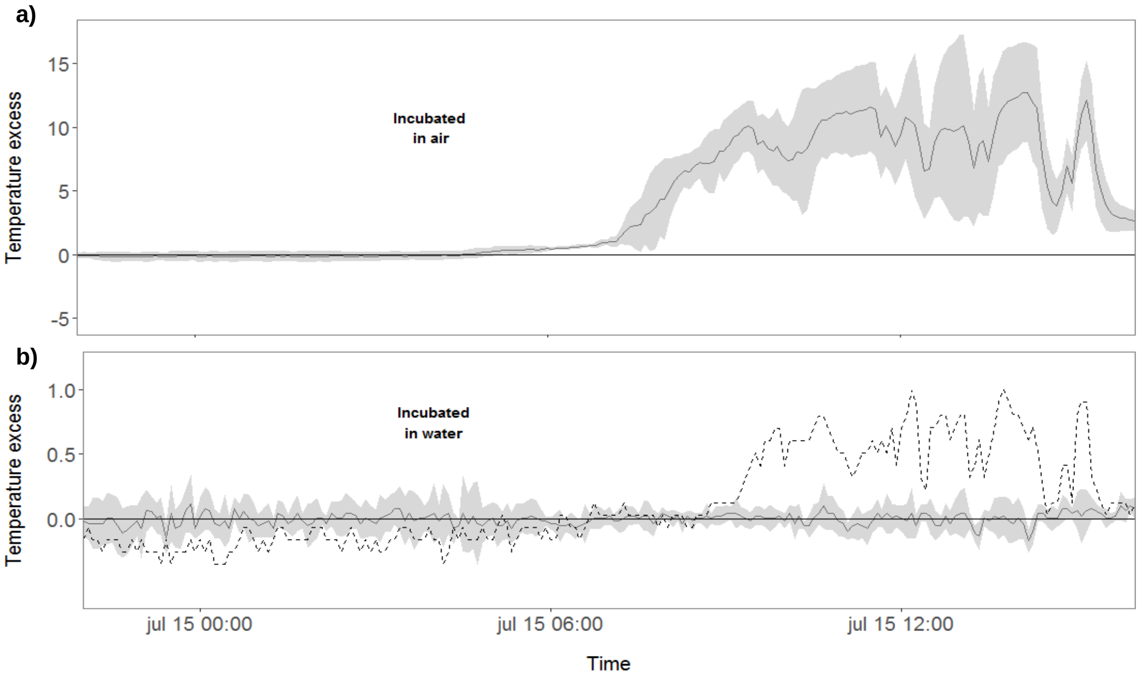

Figure S1. The effect of solar radiation on temperature excess of black compared with white objects is higher in air than in water. **a)** Daily profile illustrated by mean (solid line) ± standard deviation (shaded area) temperature excess of black compared to white data loggers incubated in air (npairs = 5), and **b)** in water (npairs = 5). The dotted line shows temperature excess of an exposed compared to a shaded logger from a separate raft.

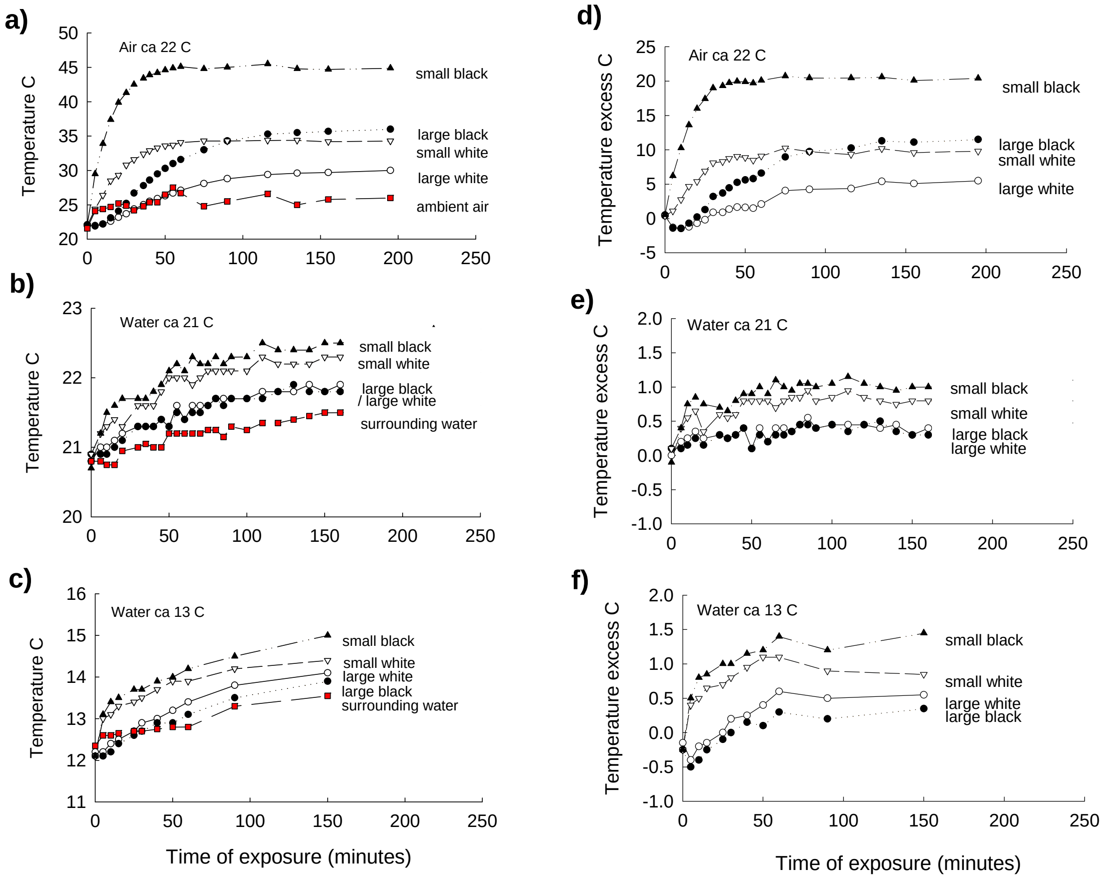

Figure S2. Effect of radiant energy on temperature of physical models depending on size, colour and medium. **a-c)** Operational core temperature (left) and **d-f)** excess temperature (right) of large and small black and white 'model fish' as a function of time exposed to artificial illumination in air (**a, d,** top panels), in warm water (**b, e,** middle panels), and in cold water (**c, f,** bottom panels).

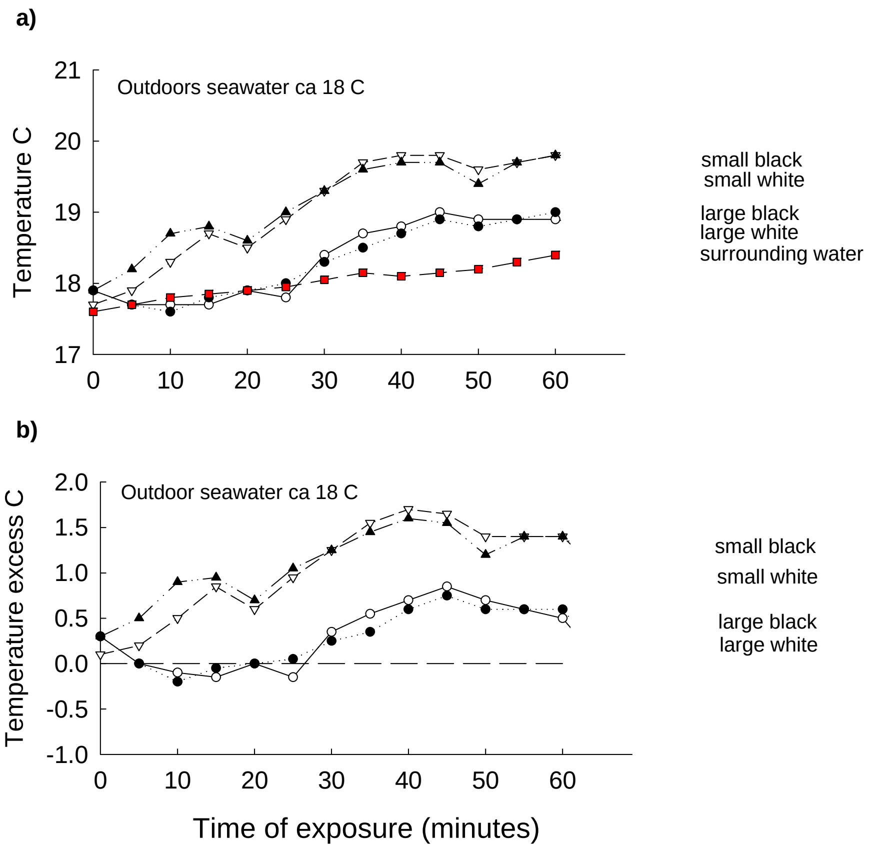

Figure S3. Temperature gain of submerged physical models exposed to solar radiation depend on size and colour. Results from comparisons of **a)** operational core temperatures and **b)** excess temperatures of physical models submerged in water as a function of time exposed to natural sunlight, during clear skies, no wind.

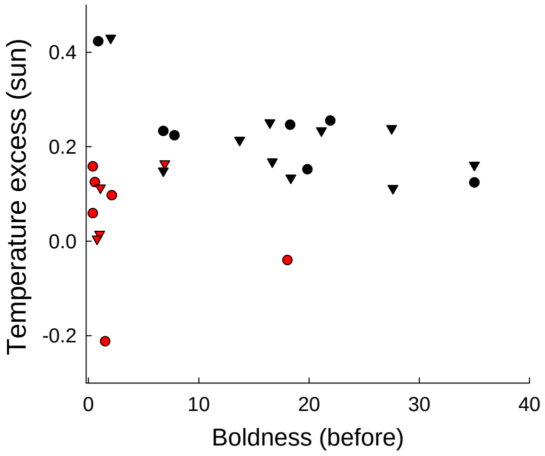

Figure S4. Associations of temperature excess with sex, colour and boldness. Temperature excess of carp during sun basking was independent on sex, higher for dark than for pale orange individuals, and increased with boldness. Figure shows mean values for individuals. Colour and shape of symbols indicate pale orange (red) and dark (black) colour morphs of female (circles) and male (triangles) carp. Results from statistical analysis are reported in Table S2.

Table S1. Associations of temperature excess in sun basking male and female carp. Results from general linear mixed model analysis of variance (GLMM) for effects of colour pattern (natural dark or pale orange), period of day (morning, midday, afternoon) and the interaction between colour and period on body temperature excess in sun basking carp. *df* represents numerator and denominator degrees of freedom. Covariance parameter estimates associated with the random effects of fish identify indicated significant variation in temperature excess among individuals both in females (covariance parameter estimate = 0.00716 ± 0.00326, $Z = 2.20$, $p = 0.0140$) and males (0.00750 ± 0.00314, $Z = 2.39$, $p = 0.0084$).

Source of variation	*df*	estimate ± SE	*F*	*P*
Females				
Colour	1, 18.6	0.187 ± 0.053	1.59	0.2233
Period of day	2, 52.8	-0.140 ± 0.103	5.17	0.0089
		-0.088 ± 0.059		
Colour x Period	2, 8173	-0.317 ± 0.089	6.41	0.0016
		-0.025 ± 0.021		
Males				
Colour	1, 19	0.246 ± 0.056	6.41	0.0203
Period of day	2, 54.4	-0.119 ± 0.108	3.02	0.0573
		-0.036 ± 0.057		
Colour x Period	2, 8883	-0.180 ± 0.082	10.81	< 0.0001
		-0.116 ± 0.025		

Table S2. Sources of variation in mean temperature excess in carp. The overall ANOVA model was significant $F_{3, 23} = 9.69$, $p = 0.0003$, $R^2 = 0.59$. Boldness represents average time to emergence from shelter box during two assessment trials before fish were released in the pond.

Source	*df*	Type III SS	Mean Square	*F*	*P*
Sex	1	0.00016275	0.00016275	0.02	0.8903
Colour	1	0.23293462	0.23293462	27.81	<.0001
Boldness	1	0.05695033	0.05695033	6.80	0.0157
